# First study on response of astrocytes in alevines of red-bellied pacu (*Piaractus brachypomus*) to subchronic exposure to chlorpyrifos and trichlorfon

**DOI:** 10.14202/vetworld.2022.1676-1683

**Published:** 2022-07-14

**Authors:** Gisella Karina Holguín-Céspedes, Ángel Enrique Céspedes-Rubio, Iang S. Rondón-Barragán

**Affiliations:** Research Group of Neurodegenerative Diseases – END, Immunotoxicology, Department of Animal Health, Faculty of Veterinary Medicine and Zootechnics, University of Tolima, Ibagué, Tolima, Colombia

**Keywords:** astrocytes, fish, neurotoxicity, organophosphates, toxicity biomarkers

## Abstract

**Background and Aim:**

Organophosphate pesticides (OPs) used in agricultural production pose environmental and public health risks whenever non-target organisms are exposed to them. Oxon-type OPs, such as trichlorfon (TCF) and chlorpyrifos (CPF), are frequently used in Colombia and have been detected in water bodies in the vicinity of croplands; however, their effect on aquatic organisms, especially fish, is largely unknown. The neurotoxicity of OPs includes inhibition of esterase enzymes, neuronal damage, and increased glial reactivity. This study aimed to assess the astrocytic response in the brain tissue of juvenile red-bellied pacu (*Piaractus brachypomus*) exposed to TCF and CPF.

**Materials and Methods:**

A 25-day subchronic assay was conducted in which juvenile red-bellied pacu were exposed to CPF and TCF. After 25 days of exposure, the fish were killed and brain samples were collected and processed for immunohistochemistry to assess the morphology and reactivity of astrocytes; glial acidic fibrillary protein was used as a biomarker.

**Results:**

The brain samples from animals under subchronic exposure to OPs for 25 days showed higher cellular density as well as changes in astrocyte phenotype characterized by shortening of cytoplasmic projections, hypertrophy, and ameboid morphology compared to those from nonexposed animals. Similarly, astrocyte hyperreactivity was detected in the optic tectum and medial longitudinal fasciculus of the exposed group.

**Conclusion:**

Immunoreactivity of brain glial cells under subchronic exposure to OPs measured through immunohistochemical tests as well as OPs-induced neuropathology may be useful as a biomarker for monitoring environmental pollution. The results also indicate that *P*. *brachypomus* is a suitable biomonitoring model for studying neurotoxicological and neurodegenerative diseases.

## Introduction

The increasing use of organophosphate pesticides (OPs) in the last several decades and the inappropriate application of these pesticides in agricultural areas have caused a number of environmental problems [1, 2]. In Colombia, agriculture is the main economic activity [3] and pesticides are frequently used to improve crop performance. Among the synthetic pesticides, oxon-type OPs, such as trichlorfon (TCF) and chlorpyrifos (CPF), are frequently used [4, 5]. Despite claims that these OPs do not persist in the environment, some studies have shown that OP residues can, in fact, persist for longer periods in organic soil and reach the drainage systems of croplands [6–8]. In Colombia, OPs can be transported to remote areas by leaching or runoff, and these toxins have been detected near and in water sources associated with crops [9], thus affecting non-target organisms [10, 11].

The toxic effect of OP exposure has been reported in terrestrial and aquatic invertebrates [12], fish [13], amphibians [14, 15], and mammals [16]. OPs affect the nervous system by inhibiting acetylcholinesterase (AChE) and other types of esterases, such as neuropathy target esterase, eliciting OP-induced delayed neuropathy syndrome [17, 18]. At the neuron level, OPs cause cellular damage accompanied by astrocyte hyperreactivity [19, 20]. Astrocytes play a pivotal role in brain homeostasis by modulating local immune responses, including those derived from cellular damage, resulting in changes in their morphology, which induces the formation of glial scars [21]. Glial fibrillary acidic protein (GFAP) is an astrocyte-specific intermediate filament protein considered a marker of astrocyte reactivity [22]. In goldfish (*Carassius auratus*), GFAP-positive glial cells have been detected in the optic tectum (OTe) and retinal fibers (Müller glia) [23]. In the zebrafish brain, GFAP was detected at the 12-somite stage [24], and the protein persists in the central nervous system of glia cells in adult zebrafish [25]. However, the astrocyte response to OP exposure in native fish species is largely unknown, and few reports have focused on astroglial response to xenobiotics [26–28].

This study aimed to assess the astrocytic response in the brain of red-bellied pacu (*Piaractus brachypomus*) in response to oxon-type OP-induced neurotoxicity, to find a molecular biomarker of environmental pollution.

## Materials and Methods

### Ethical approval

This research project was approved by the bioethics committee of the University of Tolima, under the guidelines of Law 84 (published 1989) and Resolution 8430 (published1993) for the use of living animals in experiments and following the international guidelines for the use of fish in experiments, within project code 310130517.

### Study period and location

This study was carried out from May 2017 to January 2018. Experiments were done at Laboratory of Immunology and Molecular Biology of the University of Tolima and at Laboratory of Veterinarian toxicology.

### Experimental animals

Fingerlings of red-bellied pacu, *P*. *brachypomus* (n = 72), 2 replicates for each treatment and 12 fish for each replicate, with an average body weight of 10 ± 0.5 g, were used in this study. The experiments were conducted at the Laboratory of Toxicology of the University of Tolima (Tolima, Colombia). All animals were acclimated in the laboratory for 15 days under the following conditions: Biomass density of the water ≤1 g/L [29], constant aeration without filter, water pH 6.43 ± 0.2, and temperature of 29°C ± 0.5°C. The conditions were monitored daily during the experiment. At the beginning of the acclimation period, animals were treated with sodium chloride (0.2%) to remove ectoparasites [30]. Experimental individuals were fed twice daily with a balanced commercial diet, Mojarra 32%® (Solla, Colombia), at 2% body weight per day.

### Chemicals

CPF (O,O-diethyl O-3,5,6-trichloropyridin-2-yl phosphorothioate, Dow AgroSciences, Colombia) (purity 98%), and TCF ((RS)-dimethyl(2,2,2-trichloro-1-hydroxyethyl)phosphonate, Bayer S.A., Colombia) (purity 97%) were obtained from a commercial dealer. CPF was diluted in acetone for a final concentration of 48 μg/mL. TCF was diluted in distilled water for a final concentration of 9.7 μg/mL. These stock solutions were added to the experimental units according to the concentration for each treatment.

### Exposure experiment

Red-bellied pacu fingerlings were randomly distributed into three groups, each with 24 fish. One group was exposed to 0.011 μg/L of CPF, corresponding to the 10^th^ part of the lethal concentration 50 (LC_50_) (0.11 μg/L) based on the previous experiment (unpublished data); another group was exposed to 18 μg/L of TCF based on the LC_50 (_0.18 mg/L) reported by Marín-Méndez *et al*. [30]; and a control group was not exposed to either CPF or TCF. Fingerlings were exposed for 25 days to CPF and TCF, corresponding to 10% of the lifespan of the fish, as recommended for the assessment of subchronic toxicity [31]. After the experimental period, animals were sedated using clove oil (40 mg/L, Eugeno^l®^, Proquident S.A., Antioquia, Colombia) and then sacrificed, and the brain tissues were sampled following the necropsy guidelines described by Yanong [32] and Meyers [33]. The samples were frozen at −20°C using a specimen matrix for cryostat, Tissue-Tek^®^ OCT (Sakura^®^ Finetek, USA). Brain coronal cryosections were prepared every 20 μm using a semi-automatic cryostat microtome KD-2950 (Kedee, USA). Cryosections were placed on a microscope slide and dried for 20 min; after which they were fixed with cold acetone (−20°C) for 1 min and passed through an ethanol gradient of 50%, 70%, and 90% for 2 min each and then maintained at 4°C until processing.

### Immunohistochemistry for GFAP

Brain sections were washed three times with 0.1 M phosphate buffer saline (PBS) pH 7.4 for 2 min each time. Endogenous peroxidase was then inhibited using methanol and PBS 0.1 M (1:1) with 30% hydrogen peroxide (H_2_O_2_) for 20 min. Sections were then washed three times, 2 min each time, with 0.1 M PBS and blocked at room temperature (20°C) using a blocking solution containing equine serum (1%) and 0.3% Triton X-100 diluted in 0.1 M PBS for 90 min. Afterward, sections were incubated at 20°C for 2 h with anti-GFAP primary antibody (1:500) diluted in an incubation buffer containing 0.3% bovine serum albumin and 0.3% Triton X-100 in 0.1 M PBS. After incubation, brain sections were washed three times with 0.1 M PBS, 2 min each time, and then incubated at 20°C with biotinylated secondary anti-rabbit antibody (1:250) for 2 h. The sections were washed three times again and then incubated at 20°C with an avidin-biotin-peroxidase complex (1:250 for each reagent A and B) for 1 h at half-light. The brain sections were washed three times with 0.1 M PBS and stained with 0.1% diaminobenzidine chromogen and 0.02% H_2_O_2_ diluted in 0.1 M PBS until the sections turned a light brown color. At that point, the reaction was immediately stopped with the addition of 0.1 M PBS.

Brain sections were visualized using an optical microscope (Olympus IX73P2F [Olympus, USA]), and the digital images were captured with an Olympus U-TV0.5XC-3 camera (Tokyo, Japan) paired with cellSens Standard v1.12 software (Olympus). Measurements of the changes in GFAP content between groups were conducted at 40× magnification, followed by densitometric analysis using Quantity One software v4.6.0 (Bio-Rad, USA), based on relative units of density. In addition, changes in astrocyte morphology were analyzed using Fiji-ImageJ v1.0 (SciJava, USA) [34] to measure the density, area, and perimeter of the astrocytes.

### Statistical analysis

Statistical analysis was performed using GraphPad Prism^®^ v6 for MacOS (GraphPad Software, Inc., USA). The data are presented as mean ± standard error of the mean. Comparison between groups (exposed vs. nonexposed) was performed with the Kruskal–Wallis test followed by a Dunn’s multiple comparison test, based on the validation of statistical assumptions of the test.

## Results

### Astrocyte reactivity

**Figure-1:**
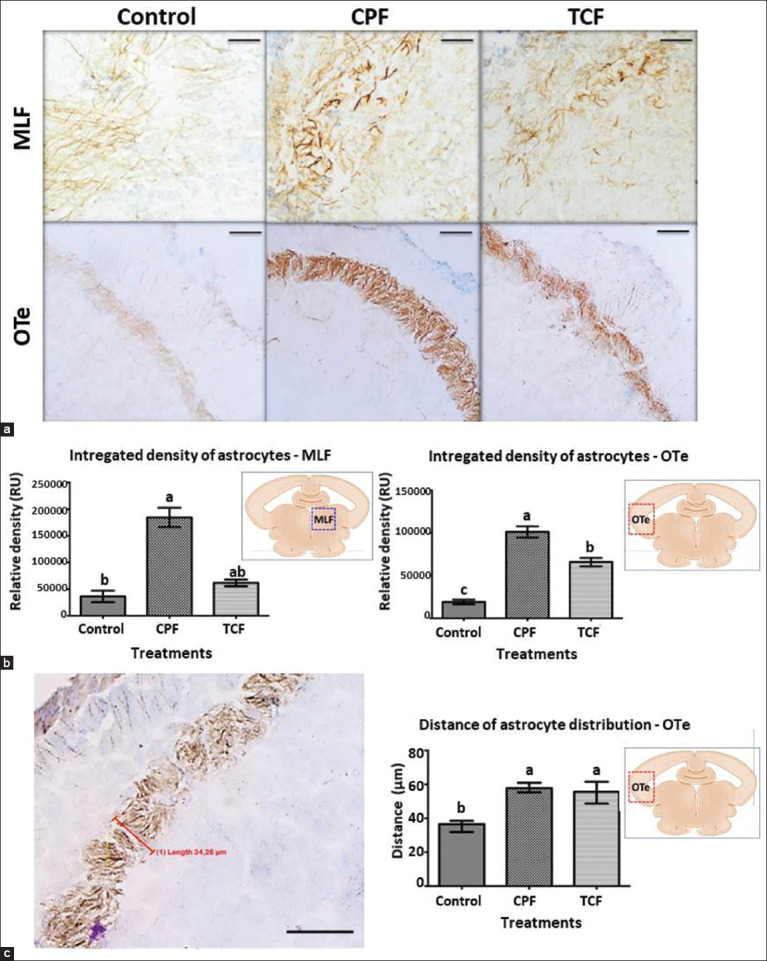
Hyperreactivity of the astroglial cells in OTe and MLF. (a) Brain coronal sections representatives from control fish and treated fish with CPF and TCF, in the OTe and MLF regions. (b) Densitometric values (Relative Units, RU) in the OTe and FLM regions (p < 0.0001). (c) Left panel, coronal section of the OTe with metric bar (red), right panel, length values (micrometers, μm) assessed in OTe (p < 0.0001). Different letters denote statistically significant differences. Scale bar = 50 μm (40×). OTe=Optic tectum, MLF = Medial longitudinal fasciculus, TCF=Trichlorfon, CPF=Chlorpyrifos.

The hyperreactivity of the astroglial cells was revealed using GFAP as a marker; density values were significantly higher (p < 0.0001) in the TCF and CPF groups compared with those in the control group in the OTe. Conversely, in the medial longitudinal fasciculus (MLF) higher density values were found only in the CPF group (p < 0.0001). The distance of distribution of the astrocytic stripe in the OTe was greater in TCF and CPF groups compared with the control group (p < 0.0001) (Figure-1).

### Morphological changes of the astrocytes

**Figure-2:**
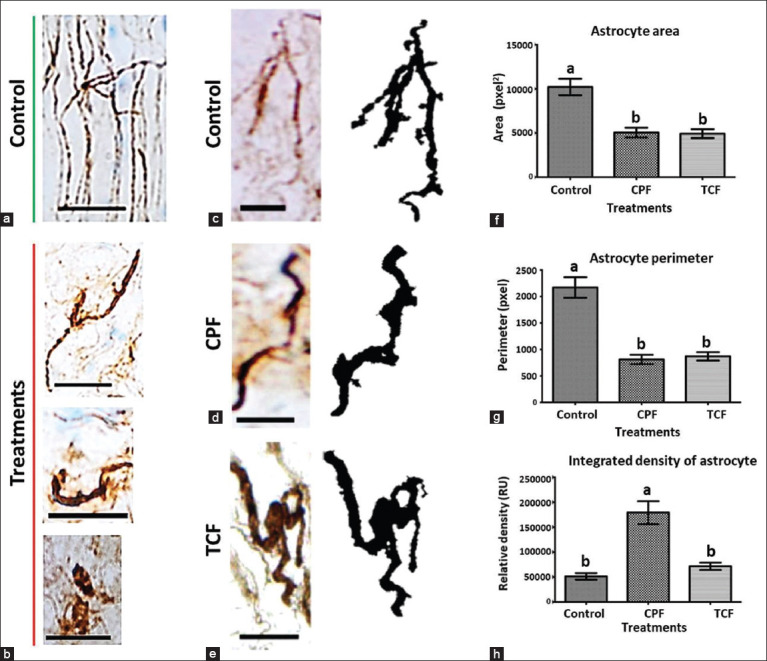
Astrocyte morphology. (a) normal radial glia (control), (b) several abnormal shapes of hyperreactive astrocytes after exposure to CPF and TCF, (c-e) photomicrographs and digitalized images (Fiji IJ) (c) normal astrocyte (Control), (d) reactive astrocyte with loss of cytoplasmic projections and thickening of the some after CPF exposure, (e) astrocyte with shortening of cytoplasmic projections and thickening of the soma. Scale = 12.5 μm. (100×). (f-h) data plots showing statistical differences in the area F and perimeter G in CPF and TCF groups versus control group, and relative density with a significant increase in CPF group (p < 0.0001). pixel=pxel, square pixel=pxel^2^. Different letters denote statistically significant differences. TCF=Trichlorfon, CPF=Chlorpyrifos.

Because of the high density in the OTe zone, it was not possible to discern morphological changes in individual astrocytes. In the MLF zone, astrocytes showed morphological changes depending on the exposure to CPF and TCF. In the control group, astrocytes appeared nonreactive having low immunoreactivity to GFAP and long, thin cytoplasmic projections were evident (Figure-2a and c). In contrast, the astrocytes of fish exposed to OPs showed high hyperreactivity characterized by hypertrophy and shortening of cytoplasmic projections as well as ameboid morphology (Figure-2b, d, and e).

The area (Figure-2f) and perimeter (Figure-2g) of the astrocytes were higher in the control group than in the CPF and TCF groups (p < 0.0001). In addition, we found an increase in the integrated density of the astrocytes in the CPF group only compared with those in the control group (Figure-2h). Individual density, expressed in relative units, increased significantly in the astrocytes of the CPF group compared with those of the control group (p < 0.001). However, no differences were found in those of the TCF group.

## Discussion

The brain is one of the main targets for OPs, and the resulting neurotoxicity appears to be due to cholinergic excitotoxicity and neuronal dysfunction [35, 36]. Changes in the cholinergic pathway have deleterious effects, including neuronal death and loss of cholinergic regions in the brain [37], even over the long term or at subclinical exposure levels in which seizures or cholinergic crisis are absent [38]. In our study, in which sublethal concentrations were applied, although there was an absence of OP-induced clinical signs, we did observe accelerated locomotion (swimming) in the exposed fish, similar to what was previously reported by Córdoba [39] and Gül [40]. In toxicity studies of guppies *Poecilia* *reticulata*, cholinergic alterations after exposure to subacute temephos and spinosad were reported [41]. Some researchers have reported temperature-dependent variation in the sensitivity to OPs [42]; however, in the present study, pH and temperature were kept constant and were suitable for the normal behavior of the studied species.

Exposure to OPs elicits neuronal damage as a result of cholinergic excitotoxicity and oxidative stress [43]. Astrocytes play an important role in the inflammatory response triggered by tissue damage in the central nervous system [44]. Their susceptibility to OP exposure has been described in the previous studies [45, 46]. In the present study, an increase in astrocyte density was found in the OTe region in response to treatments with CPF (0.011 μg/L) and TCF (0.18 μg/L) and in the MLF region under CPF exposure. Similarly, an increase in the distance of astrocyte distribution was evident. Increases in astrocyte density due to subchronic OP-induced toxicity have been reported in murine models, in which GFAP was used as a marker of reactivity [47], similar to the findings of the present study. This increase in reactivity has been associated with the neuroprotective role of glial cells against OP toxicity [46] and can be a consequence of the inhibition of neuropathy target esterase [48], eliciting an imbalance in the levels of lysophosphatidylcholine in the neuron cell membrane, which, in turn, brings about cytotoxicity, characterized by chronic demyelination [49, 50].

Because of demyelination processes, healing mechanisms are activated [51], and astrocytes play a role in phagocytosis and the removal of myelin debris, as well as the release of cytokines, namely, Interleukin (IL)-1, IL-6, Interferon-gamma, and tumor necrosis factor-alpha, thus contributing to a general inflammatory reaction and astrocyte proliferation [52]. This process might explain the increase in astrocytic density in the experimental groups exposed to toxins.

Although some investigators have described the OTe region as immunonegative to GFAP [53], we found in the present experiment that GFAP+-astrocytes in the OTe region of animals exposed to OPs were ameboid in shape and had higher reactivity to the toxin. Bignami and Dahl [54] described low reactivity of GFAP+-astrocytes in the same region from an intact fish brain, and Nielsen and Jørgensen [55] reported no changes in GFAP+-astrocytes in the OTe region after mechanical injury. Kálmán *et al*. [53] hypothesized that the lack of GFAP reactivity can be attributed to the lack of astroglial elements that would normally synthesize GFAP. In our study, we found GFAP+-astrocytes in the control and exposed groups, as well as an increase in the GFAP reactivity after exposure to OPs, which allows us to infer that GFAP is a constitutive protein of astrocytes in this species and detectable using immunohistochemical methods.

GFAP reactivity in mammals is similar to the reactivity in red-bellied pacu lesions in our study, which further supports our proposal that this fish species can be utilized as a model to evaluate toxic or degenerative neurological pathologies. Lee *et al*. [56] also showed a high degree of similarity between fish and mammals at the genetic and genomic level with respect to the production of GFAP, and highlighted the potential value of piscine models in the study of mutation pathogenesis, in particular Alexander’s disease.

Exposure to OPs in red-bellied pacu induced astrocytic changes in the evaluated brain regions; however, in the case of CPF, a higher reactivity was found in the MLF, which was not the case in the exposure to TCF. The different metabolites of the two OPs might explain this result; trichloropyridinol (from CPF) can induce higher damage to the astrocytes compared with metabolites from other OPs (e.g., dichlorvos from TCF) [46]. However, it is still unknown if this difference in reactivity is based on more rapid diffusion of the metabolite into the cell population or the higher toxicity of the metabolite itself. Ozawa *et al*. [57] reported that the exposure to dichlorvos can affect the production of GFAP in the differentiation of the astrocytes through the inhibition of AChE, which may explain the differences in response to OPs; nevertheless, this mechanism has been found in C6 cells from murine glioma but not in fish. The damage of astrocyte populations may exacerbate the neuronal damage induced by OPs.

Astrocytes at the injury site play a pivotal role in the homeostasis of the extracellular milieu, favoring neuronal survival. However, astrocyte hyperreactivity over the long term may generate a glial scar, which impairs the neuronal interconnections and the synaptic transmission [58, 59]. The process of glial scarring includes an increase of the length, density, and area of the astrocytes, characteristic of astrocyte reactivity [60], as well as a shortening of the cytoplasmic projections, hypertrophy, and condensation of the cell soma [61], which were features evident in the fish exposed to CPF and TCF in our study and can be indicative of injury and astrocyte response induced by toxins.

The role of glial cells in the immune response of the central nervous system and the variability of this response under toxic exposure (e.g., CPF and TCF) has been reported by Griffiths *et al*. [27]. In fish, the mechanisms involved in the regulation of glial responses to neurotoxic agents are still unknown. In goldfish (*C. auratus*), glial cells positive to GFAP have been reported by Nona *et al*. [26], and other studies have revealed the abnormal formation of cell membranes of glial cells from fish in response to injury [62]; however, to the best of our knowledge, the present study is the first report of the variation in astrocytes in response to OPs.

Zebrafish (*Danio rerio*) has been used as a model for monitoring the central nervous system *in vivo* [63, 64], and other species of teleosts have been studied for changes in behavior and learning processes after exposure to CPF [65]. However, our study is the first that is designed with the goal of understanding the mechanisms of astrocyte response against exposure to oxon-type OPs in fish and the first evidence-based proposal for an alternative to environmental screening, that is, the use of *P*. *brachypomus* as an animal model in the study of neurodegenerative diseases that affects humans.

## Conclusion

The immunoreactivity of brain glial cells after exposure to CPF and TCF in a subchronic phase may be considered a feasible test for assessing environmental pollution, and we have shown with this study that this assessment can be accomplished using immunohistochemical biomarkers in fish. Morphological changes in glial cells, such as shortening of the cytoplasmic projections, hypertrophy, and condensation of cell soma, are indicative of glial reactivity in *P*. *brachypomus* after exposure to CPF and TCF. These results allow us to characterize the neuropathological and neurotoxicological lesions induced in the experimental model of *P*. *brachypomus*, which can be used as a model for the analogic study of neurodegenerative and neurotoxicological diseases. Further studies are necessary to determine astrocyte changes, as well as other cellular lineages such as oligodendrocytes, astrocytes, microglia, dendritic cells at different experimental conditions (e.g. time, concentration, recovery) and to perform biochemical studies that focus on the expression of pro- inflammatory cytokines and proteins that modulate the response against pesticides, in particular OPs.

## Authors’ Contributions

AECR and ISR: Designed the study. GKH: Performed the experiments and laboratory analyses. AECR: Administered the project. ISR and GKH: Wrote the manuscript. ISR, GKH, and AECR: Reviewed and edited the manuscript. ISR and AECR: Revised the manuscript critically. All authors have read and approved the final manuscript.
